# Prosecutions of Medical Men

**Published:** 1853-10

**Authors:** 


*Prosecutions of Medical Men.*—Within the past year several suits have been commenced and carried through against medical men for malpractice. Among those in this vicinity we may mention the trials of Dr. Hammond, of Nashua, and Dr. Sargent, of Rochester in this State, and more recently that of Dr. Kittredge, of Andover, Massachusetts. In the first case, Dr. Hammond was acquitted, not more in consequence of the ability of his counsel than the honesty and independence of the surgeon called to testify for the plaintiff. In Dr. Sargent's case we are informed that the verdict was given for the plaintiff in the face of the most explicit testimony from medical men. The same was true in Dr. Kittredge's trial, in which, as we understand it, after an injury to the arm in which there was rupture of the brachial artery, the at tending surgeon was brought in guilty for causing the arm to slough off by tight bandaging. The community should be made to understand that by encouraging such prosecutions they are endangering their own safety, and surgeons will be compelled in self-defence to require beforehand a bond that they shall not be prosecuted, whatever may be the result of the treatment. From several recent trials we feel warranted in saying that the chances are altogether better for the acquittal of an ignorant, uneducated pretender to medical knowledge, who is really guilty, than for that of an intelligent, well-educated surgeon to whom no fault can justly be charged.—*New Hampshire Jour. of Medicine.*

